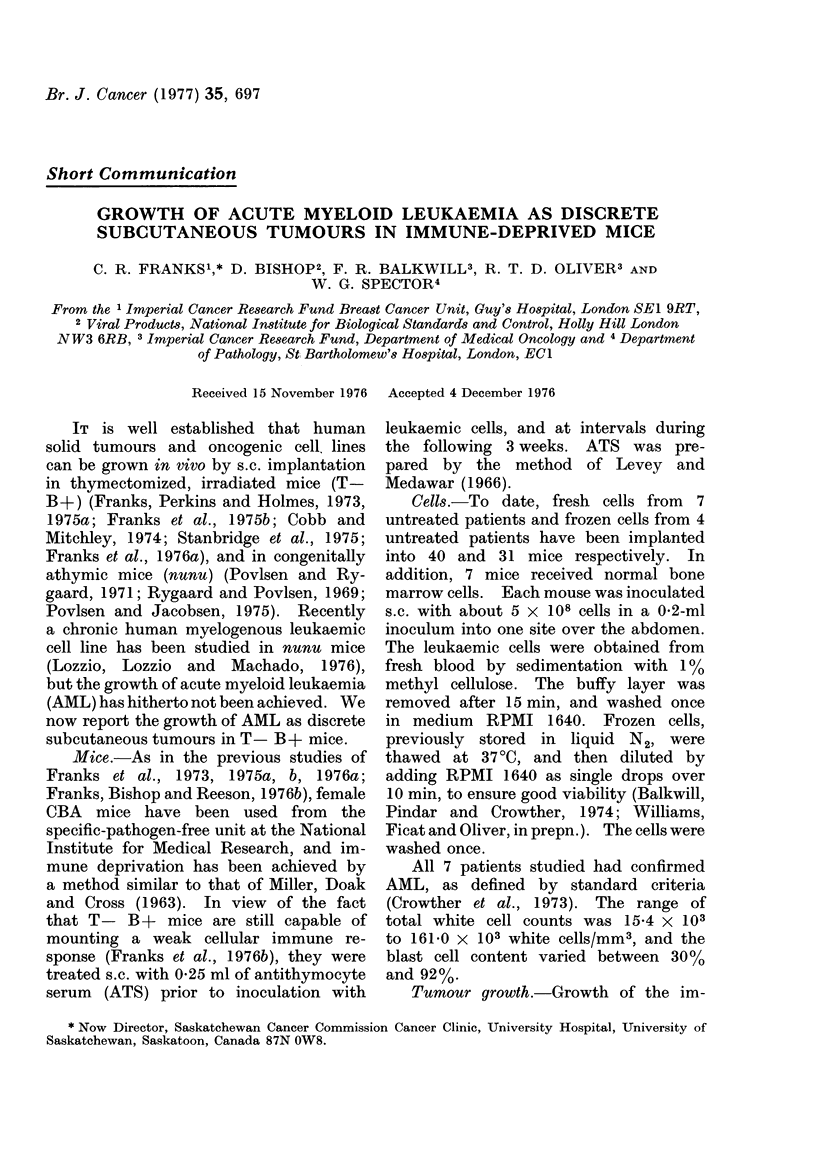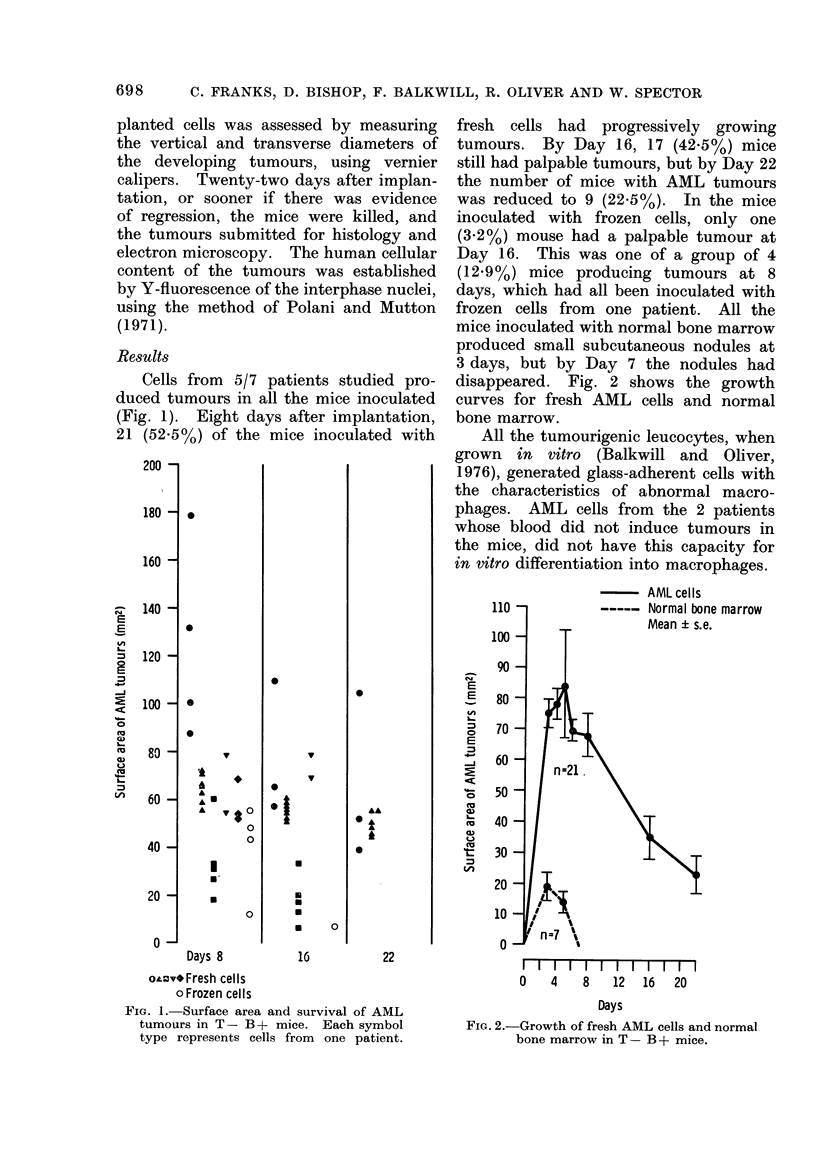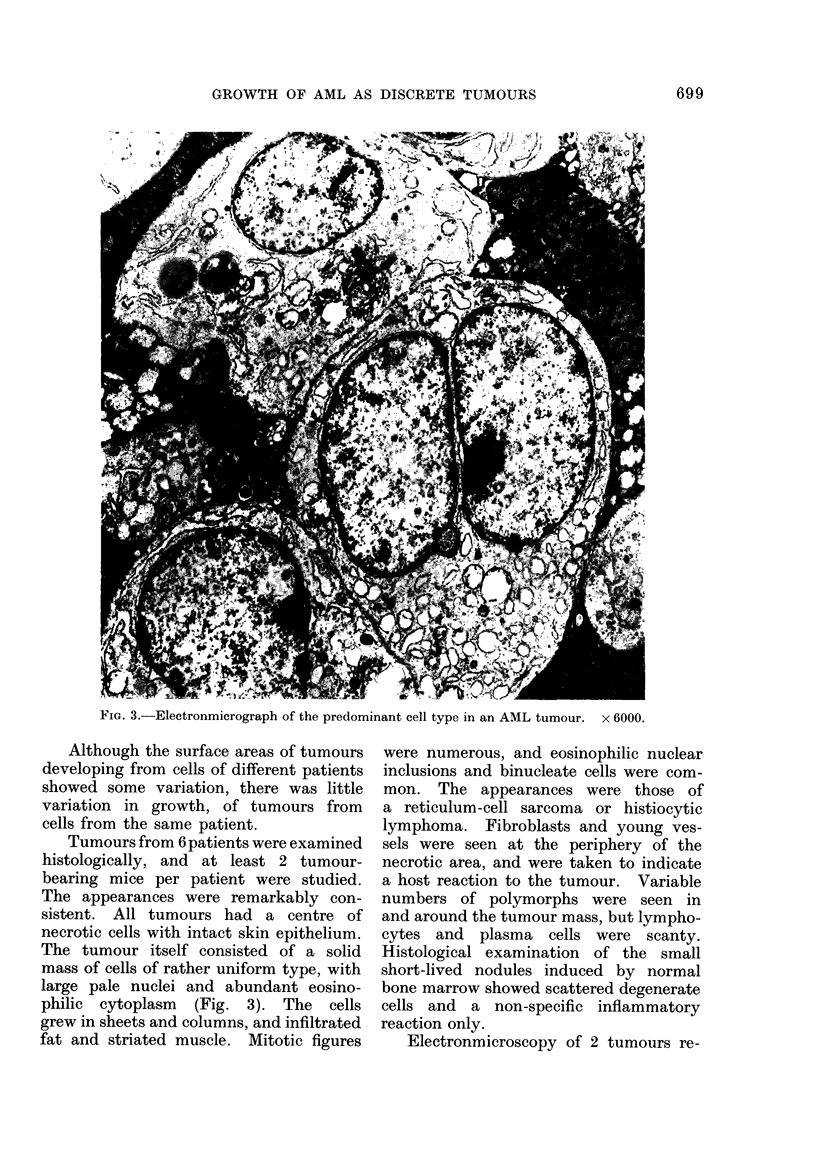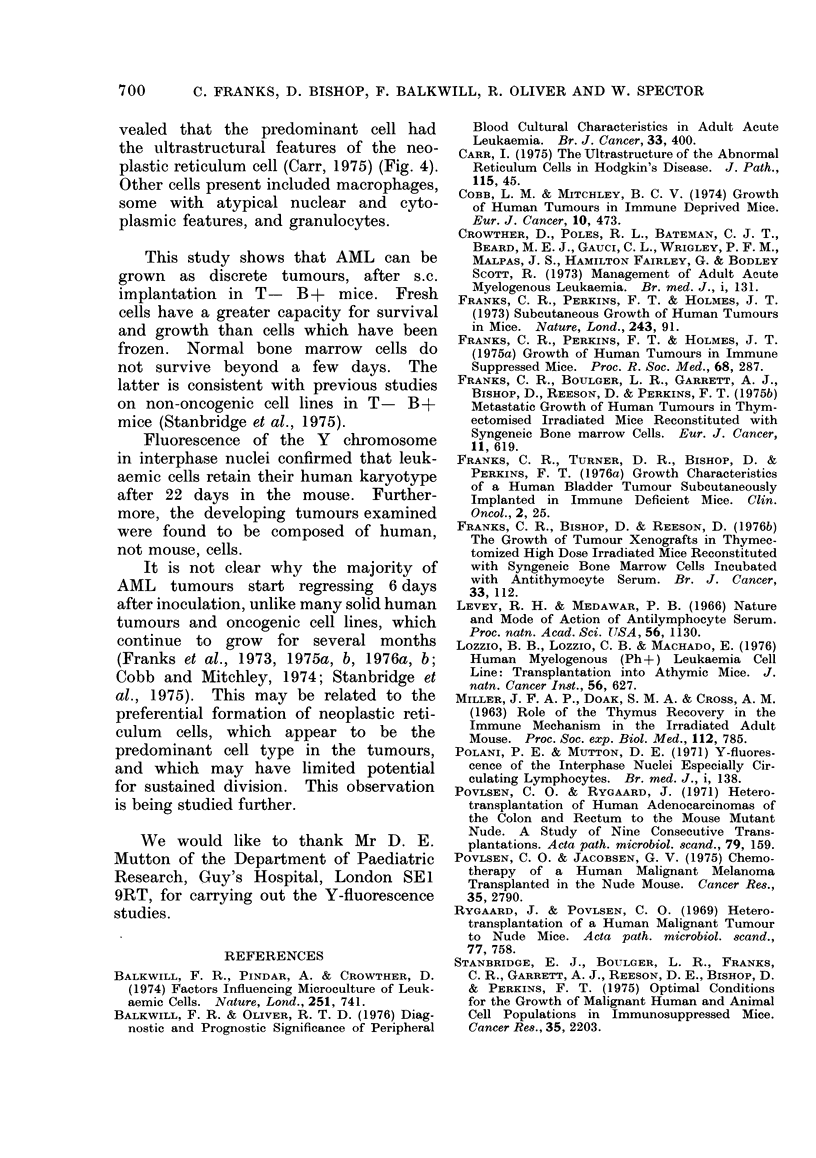# Growth of acute myeloid leukaemia as discrete subcutaneous tumours in immune-deprived mice.

**DOI:** 10.1038/bjc.1977.107

**Published:** 1977-05

**Authors:** C. R. Franks, D. Bishop, F. R. Balkwill, R. T. Oliver, W. G. Spector

## Abstract

**Images:**


Br. J. Cancer (1977) 35, 697

*Short Communication*

### GROWTH OF ACUTE MYELOID LEUKAEMIA AS DISCRETE SUBCUTANEOUS TUMOURS IN IMMUNE-DEPRIVED MICE

C. R. FRANKS[1],* D. BISHOP[2], F. R. BALKWILL[3], R. T. D. OLIVER[3] AND W. G. SPECTOR[4]

*From the [1] Imperial Cancer Research Fund Breast Cancer Unit, Guy's Hospital, London SE1 9RT, [2] Viral Products, National Institute for Biological Standards and Control, Holly Hill London NW3 6RB, [3] Imperial Cancer Research Fund, Department of Medical Oncology and [4] Department of Pathology, St Bartholomew's Hospital, London, EC1*

Received 15 November 1976 Accepted 4 December 1976

IT is well established that human solid tumours and oncogenic cell lines can be grown *in vivo* by s.c. implantation in thymectomized, irradiated mice (T— B+) (Franks, Perkins and Holmes, 1973, 1975*a*; Franks *et al.*, 1975*b*; Cobb and Mitchley, 1974; Stanbridge *et al.*, 1975; Franks *et al.*, 1976*a*), and in congenitally athymic mice (*nunu*) (Povlsen and Rygaard, 1971; Rygaard and Povlsen, 1969; Povlsen and Jacobsen, 1975). Recently a chronic human myelogenous leukaemic cell line has been studied in *nunu* mice (Lozzio, Lozzio and Machado, 1976), but the growth of acute myeloid leukaemia (AML) has hitherto not been achieved. We now report the growth of AML as discrete subcutaneous tumours in T— B+ mice.

*Mice.*—As in the previous studies of Franks *et al.*, 1973, 1975*a*, *b*, 1976*a*; Franks, Bishop and Reeson, 1976*b*), female CBA mice have been used from the specific-pathogen-free unit at the National Institute for Medical Research, and immune deprivation has been achieved by a method similar to that of Miller, Doak and Cross (1963). In view of the fact that T— B+ mice are still capable of mounting a weak cellular immune response (Franks *et al.*, 1976*b*), they were treated s.c. with 0·25 ml of antithymocyte serum (ATS) prior to inoculation with leukaemic cells, and at intervals during the following 3 weeks. ATS was prepared by the method of Levey and Medawar (1966).

*Cells.*—To date, fresh cells from 7 untreated patients and frozen cells from 4 untreated patients have been implanted into 40 and 31 mice respectively. In addition, 7 mice received normal bone marrow cells. Each mouse was inoculated s.c. with about $5 \times 10^8$ cells in a 0·2-ml inoculum into one site over the abdomen. The leukaemic cells were obtained from fresh blood by sedimentation with 1% methyl cellulose. The buffy layer was removed after 15 min, and washed once in medium RPMI 1640. Frozen cells, previously stored in liquid $N_2$, were thawed at 37°C, and then diluted by adding RPMI 1640 as single drops over 10 min, to ensure good viability (Balkwill, Pindar and Crowther, 1974; Williams, Ficat and Oliver, in prepn.). The cells were washed once.

All 7 patients studied had confirmed AML, as defined by standard criteria (Crowther *et al.*, 1973). The range of total white cell counts was $15{\cdot}4 \times 10^3$ to $161{\cdot}0 \times 10^3$ white cells/mm$^3$, and the blast cell content varied between 30% and 92%.

*Tumour growth.*—Growth of the im-

* Now Director, Saskatchewan Cancer Commission Cancer Clinic, University Hospital, University of Saskatchewan, Saskatoon, Canada 87N 0W8.

planted cells was assessed by measuring the vertical and transverse diameters of the developing tumours, using vernier calipers. Twenty-two days after implantation, or sooner if there was evidence of regression, the mice were killed, and the tumours submitted for histology and electron microscopy. The human cellular content of the tumours was established by Y-fluorescence of the interphase nuclei, using the method of Polani and Mutton (1971).

*Results*

Cells from 5/7 patients studied produced tumours in all the mice inoculated (Fig. 1). Eight days after implantation, 21 (52·5%) of the mice inoculated with fresh cells had progressively growing tumours. By Day 16, 17 (42·5%) mice still had palpable tumours, but by Day 22 the number of mice with AML tumours was reduced to 9 (22·5%). In the mice inoculated with frozen cells, only one (3·2%) mouse had a palpable tumour at Day 16. This was one of a group of 4 (12·9%) mice producing tumours at 8 days, which had all been inoculated with frozen cells from one patient. All the mice inoculated with normal bone marrow produced small subcutaneous nodules at 3 days, but by Day 7 the nodules had disappeared. Fig. 2 shows the growth curves for fresh AML cells and normal bone marrow.

All the tumourigenic leucocytes, when grown *in vitro* (Balkwill and Oliver, 1976), generated glass-adherent cells with the characteristics of abnormal macrophages. AML cells from the 2 patients whose blood did not induce tumours in the mice, did not have this capacity for *in vitro* differentiation into macrophages.

FIG. 1.—Surface area and survival of AML tumours in T— B+ mice. Each symbol type represents cells from one patient.

FIG. 2.—Growth of fresh AML cells and normal bone marrow in T— B+ mice.

FIG. 3.—Electronmicrograph of the predominant cell type in an AML tumour. ×6000.

Although the surface areas of tumours developing from cells of different patients showed some variation, there was little variation in growth, of tumours from cells from the same patient.

Tumours from 6 patients were examined histologically, and at least 2 tumour-bearing mice per patient were studied. The appearances were remarkably consistent. All tumours had a centre of necrotic cells with intact skin epithelium. The tumour itself consisted of a solid mass of cells of rather uniform type, with large pale nuclei and abundant eosinophilic cytoplasm (Fig. 3). The cells grew in sheets and columns, and infiltrated fat and striated muscle. Mitotic figures were numerous, and eosinophilic nuclear inclusions and binucleate cells were common. The appearances were those of a reticulum-cell sarcoma or histiocytic lymphoma. Fibroblasts and young vessels were seen at the periphery of the necrotic area, and were taken to indicate a host reaction to the tumour. Variable numbers of polymorphs were seen in and around the tumour mass, but lymphocytes and plasma cells were scanty. Histological examination of the small short-lived nodules induced by normal bone marrow showed scattered degenerate cells and a non-specific inflammatory reaction only.

Electronmicroscopy of 2 tumours revealed that the predominant cell had the ultrastructural features of the neoplastic reticulum cell (Carr, 1975) (Fig. 4). Other cells present included macrophages, some with atypical nuclear and cytoplasmic features, and granulocytes.

This study shows that AML can be grown as discrete tumours, after s.c. implantation in T− B+ mice. Fresh cells have a greater capacity for survival and growth than cells which have been frozen. Normal bone marrow cells do not survive beyond a few days. The latter is consistent with previous studies on non-oncogenic cell lines in T− B+ mice (Stanbridge *et al.*, 1975).

Fluorescence of the Y chromosome in interphase nuclei confirmed that leukaemic cells retain their human karyotype after 22 days in the mouse. Furthermore, the developing tumours examined were found to be composed of human, not mouse, cells.

It is not clear why the majority of AML tumours start regressing 6 days after inoculation, unlike many solid human tumours and oncogenic cell lines, which continue to grow for several months (Franks *et al.*, 1973, 1975*a*, *b*, 1976*a*, *b*; Cobb and Mitchley, 1974; Stanbridge *et al.*, 1975). This may be related to the preferential formation of neoplastic reticulum cells, which appear to be the predominant cell type in the tumours, and which may have limited potential for sustained division. This observation is being studied further.

We would like to thank Mr D. E. Mutton of the Department of Paediatric Research, Guy's Hospital, London SE1 9RT, for carrying out the Y-fluorescence studies.

### REFERENCES

Balkwill, F. R., Pindar, A. & Crowther, D. (1974) Factors Influencing Microculture of Leukaemic Cells. *Nature, Lond.*, **251**, 741.

Balkwill, F. R. & Oliver, R. T. D. (1976) Diagnostic and Prognostic Significance of Peripheral Blood Cultural Characteristics in Adult Acute Leukaemia. *Br. J. Cancer*, **33**, 400.

Carr, I. (1975) The Ultrastructure of the Abnormal Reticulum Cells in Hodgkin's Disease. *J. Path.*, **115**, 45.

Cobb, L. M. & Mitchley, B. C. V. (1974) Growth of Human Tumours in Immune Deprived Mice. *Eur. J. Cancer*, **10**, 473.

Crowther, D., Poles, R. L., Bateman, C. J. T., Beard, M. E. J., Gauci, C. L., Wrigley, P. F. M., Malpas, J. S., Hamilton Fairley, G. & Bodley Scott, R. (1973) Management of Adult Acute Myelogenous Leukaemia. *Br. med. J.*, i, 131.

Franks, C. R., Perkins, F. T. & Holmes, J. T. (1973) Subcutaneous Growth of Human Tumours in Mice. *Nature, Lond.*, **243**, 91.

Franks, C. R., Perkins, F. T. & Holmes, J. T. (1975*a*) Growth of Human Tumours in Immune Suppressed Mice. *Proc. R. Soc. Med.*, **68**, 287.

Franks, C. R., Boulger, L. R., Garrett, A. J., Bishop, D., Reeson, D. & Perkins, F. T. (1975*b*) Metastatic Growth of Human Tumours in Thymectomised Irradiated Mice Reconstituted with Syngeneic Bone marrow Cells. *Eur. J. Cancer*, **11**, 619.

Franks, C. R., Turner, D. R., Bishop, D. & Perkins, F. T. (1976*a*) Growth Characteristics of a Human Bladder Tumour Subcutaneously Implanted in Immune Deficient Mice. *Clin. Oncol.*, **2**, 25.

Franks, C. R., Bishop, D. & Reeson, D. (1976*b*) The Growth of Tumour Xenografts in Thymectomized High Dose Irradiated Mice Reconstituted with Syngeneic Bone Marrow Cells Incubated with Antithymocyte Serum. *Br. J. Cancer*, **33**, 112.

Levey, R. H. & Medawar, P. B. (1966) Nature and Mode of Action of Antilymphocyte Serum. *Proc. natn. Acad. Sci. USA*, **56**, 1130.

Lozzio, B. B., Lozzio, C. B. & Machado, E. (1976) Human Myelogenous (Ph+) Leukaemia Cell Line: Transplantation into Athymic Mice. *J. natn. Cancer Inst.*, **56**, 627.

Miller, J. F. A. P., Doak, S. M. A. & Cross, A. M. (1963) Role of the Thymus Recovery in the Immune Mechanism in the Irradiated Adult Mouse. *Proc. Soc. exp. Biol. Med.*, **112**, 785.

Polani, P. E. & Mutton, D. E. (1971) Y-fluorescence of the Interphase Nuclei Especially Circulating Lymphocytes. *Br. med. J.*, i, 138.

Povlsen, C. O. & Rygaard, J. (1971) Heterotransplantation of Human Adenocarcinomas of the Colon and Rectum to the Mouse Mutant Nude. A Study of Nine Consecutive Transplantations. *Acta path. microbiol. scand.*, **79**, 159.

Povlsen, C. O. & Jacobsen, G. V. (1975) Chemotherapy of a Human Malignant Melanoma Transplanted in the Nude Mouse. *Cancer Res.*, **35**, 2790.

Rygaard, J. & Povlsen, C. O. (1969) Heterotransplantation of a Human Malignant Tumour to Nude Mice. *Acta path. microbiol. scand.*, **77**, 758.

Stanbridge, E. J., Boulger, L. R., Franks, C. R., Garrett, A. J., Reeson, D. E., Bishop, D. & Perkins, F. T. (1975) Optimal Conditions for the Growth of Malignant Human and Animal Cell Populations in Immunosuppressed Mice. *Cancer Res.*, **35**, 2203.